# Intranasal Immunization with Nasal Immuno-Inducible Sequence-Fused Antigens Elicits Antigen-Specific Antibody Production

**DOI:** 10.3390/ijms252312828

**Published:** 2024-11-28

**Authors:** Hiraku Sasaki, Yoshio Suzuki, Kodai Morimoto, Kazuyoshi Takeda, Koichiro Uchida, Masayuki Iyoda, Hiroki Ishikawa

**Affiliations:** 1 Graduate School of Health and Sports Science, Juntendo University, Chiba 2701695, Japan; yssuzuki@juntendo.ac.jp; 2 Center for Immune Therapeutics and Diagnosis, Juntendo University, Tokyo 1138421, Japan; k.morimoto.fm@juntendo.ac.jp (K.M.); ktakeda@juntendo.ac.jp (K.T.); k-uchida@juntendo.ac.jp (K.U.); 3 Department of Biofunctional Microbiota, Graduate School of Medicine, Juntendo University, Tokyo 1138421, Japan; 4 Laboratory of Cell Biology, Research Support Center, Graduate School of Medicine, Juntendo University, Tokyo 1138421, Japan; 5 Department of Microbiology and Immunology, Showa University School of Medicine, Tokyo 1428555, Japan; iyoda@med.showa-u.ac.jp (M.I.); h_ishikawa@med.showa-u.ac.jp (H.I.)

**Keywords:** intranasal immunization, intranasal vaccine, nasal immune-inducible sequence

## Abstract

Intranasal immunization is one of the most effective methods for eliciting lung mucosal immunity. Multiple intranasal immunization with bacterial polypeptide, termed as a modified PnxIIIA (MP3) protein, is known to elicit production of a specific antibody in mice. In this study, a nasal immuno-inducible sequence (NAIS) was designed to remove the antigenicity of the MP3 protein that can induce mucosal immunity by intranasal immunization, and was examined to induce antigen-specific antibodies against the fused bacterial thioredoxin (Trx) as a model antigen. A NAIS was modified and generated to remove a large number of predicted MHC (Major Histocompatibility Complex)-I and MHC-II binding sites in parent protein PnxIIIA and MP3 in order to reduce the number of antigen epitope sites. For comparative analysis, full-length NAIS291, NAIS230, and NAIS61 fused with Trx and 6× His tag and Trx-fused 6× His tag were used as antigen variants for the intranasal immunization of BALB/c mice every two weeks for three immunizations. Anti-Trx antibody titers in serum and bronchoalveolar lavage fluid (BALF) IgA obtained from NAIS291-fused Trx-immunized mice were significantly higher than those from Trx-immunized mice. The antibody titers against NAIS alone were significantly lower than those against Trx alone in the serum IgG, serum IgA, and BALF IgA. These results indicate that the NAIS contributes to antibody elicitation of the fused antigen as an immunostimulant in intranasal vaccination vaccines. The results indicate that the NAIS and target inactivated antigen fusions can be applied to intranasal vaccine systems.

## 1. Introduction

Intranasal immunization is one of the most effective methods for inducing mucosal immunity, without the need for special equipment. Intranasal immunization vaccines are easily administered without needles. After T cells are activated by antigen presentation from antigen presenting cells (APCs) in nasal-associated lymphoid tissue (NALT), B cells differentiate into IgA plasma cells and induce secretory IgA (sIgA) production, which can prevent pathogen entry [1,2]. The sIgA obtained from intranasal vaccines plays an essential role in infection defense because it eliminates the invading pathogen itself. It effectively prevents pathogen entry through the upper respiratory tract and provides strong immunity against pathogens that invade with systemic antibodies, including organs such as the vagina and intestinal tract [1,2]. Thus, intranasal vaccines are the most effective for protecting against infection if they function consistently.

Although intranasal vaccines generally use live attenuated vaccines and immunization restrictions are imposed for safety reasons [3,4], using inactivated vaccines can improve vaccine safety and expand the vaccination target range [5,6]. Bacterial toxins, including cholera and heat-labile *Escherichia coli* toxin, have long been used as experimental immunostimulants for intranasal vaccines; however, their safety is yet to be secured adequately [7]. In addition, live viruses, polymers, and plant saponins have been devised as adjuvant alternatives or a medium for transport systems and immune induction; however, whether all carriers are appropriate for intranasal forms of inoculation or even for multiple vaccinations is not fully clear [8,9,10]. All these systems have advantages, but further improvements may be necessary to increase their safety and antigenicity in experimental and practical applications.

Since adjuvants or immunomodulators inoculated with antigens play important roles during the intranasal vaccination of an inactivated candidate, we developed an immunomodulatory bacterial outer membrane protein PnxIIIA with low cytotoxicity and extracellular matrix adhesion properties from the genus *Rodentibacter* [11,12,13]. Although this protein is a member of the RTX (repeat in structural toxin) toxin family of pore-forming toxins secreted by the bacterial type I secretion machinery found in *Pasteurellaceae*, it is less toxic than the previously found PnxIA, PnxIIA, and other RTX toxin members, and is specialized for extracellular matrix adhesion in genus *Rodentibacter* [12,13]. Of the RTX toxins produced by *Rodentibacter*, PnxIIIA is a high molecular weight protein of 250 kda that is highly immunostimulatory to host cell responses and is solubilized and secreted from the outer membrane of *Rodentibacter* species and *E. coli* [13]. Therefore, PnxIIIA is also highly immunogenic, can be easily expressed in the *E. coli* expression system, and is a candidate protein that was initially conceived as an adhesive mucosal vaccine model for genus *Rodentibacter* [11,13]. Using PnxIIIA protein modified by removing the toxin domain, non-adjuvanted recombinant modified PnxIIIA (MP3) protein elicited specific serum IgG and sIgA responses after three or more intranasal vaccinations in a mouse study, and experimental infections of the parent *R. pneumotropicus* showed that even opportunistic infections were defensible in mice [11]. Therefore, MP3 proteins lacking antigenicity of MP3 fused with target antigens may be used as immunomodulators in intranasal vaccines. If inactivated vaccine antigens can be fused to this MP3 protein and induce antibodies as in the previous study simply by intranasal inoculation, it could be applied to any pathogen and be a paradigm shift in the vaccine system. To verify their efficacy, the antigenicity of MP3 proteins should be reduced and animal experiments using antigen-fused proteins should be conducted.

In this study, we re-designed MP3 protein as a nasal immune-inducible sequence (NAIS) for intranasal vaccines and examined whether it can be used as an immunomodulator for intranasal vaccine. For the target antigen, we used the *E. coli* thioredoxin (Trx), which is also present in all organisms and is easily produced stably in a prokaryotic cell expression system as a model antigen.

## 2. Results

### 2.1. Vaccine Design and Cytotoxicity

**Figure 1:**
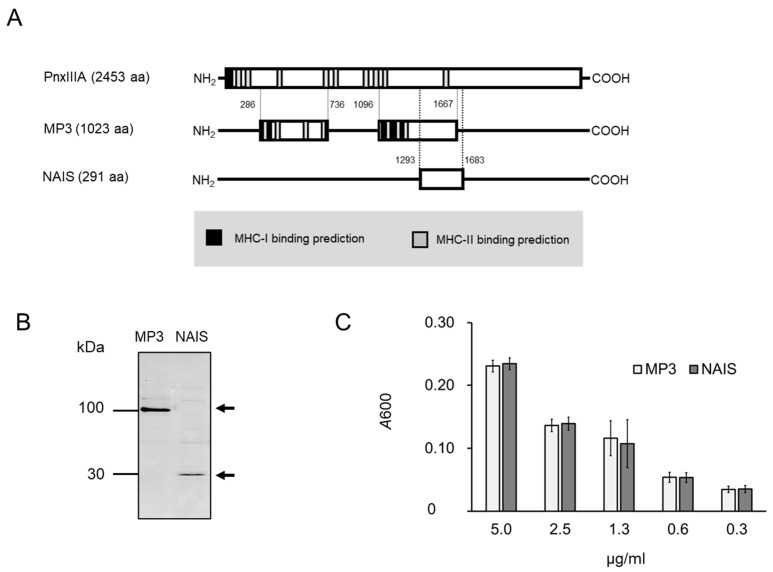
(**A**) Schematic representation of primary structure of PnxIIIA [13], modified PnxIIIA (MP3) [11] and nasal immuno-inducible sequence (NAIS), and binding sites for MHC-I and MHC-II that were predicted by the immune epitope database (IEDB, https://www.iedb.org/ accessed on 25 November 2024) [14,15] in PnxIIIA and MP3. MHC-I and MHC-II binding sites shown relatively summarize the top 50 predicted affinity sites. PnxIIIA in figure is based on GenBank accession number BAJ09609.1, and numbers listed are shown with reference to the amino acid sequence of PnxIIIA. Figure is not to scale. (**B**) SDS-PAGE analysis of MP3 and NAIS. (**C**) Comparison of collagen type IV binding ability between MP3 and NAIS. There were no significant differences between MP3 and NAIS in collagen-binding ability (*p* ≥ 0.05).

**Figure 2:**
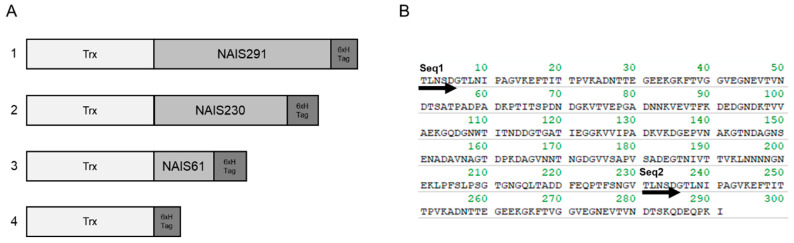
Schematic representation of (**A**) primary structure of intranasal immunized materials and (**B**) amino acid sequence of nasal immune-inducible sequence (NAIS). In (**A**), Trx and 6× H tag indicates the thioredoxin protein and 6× His tag, respectively. 1: 291, 2: 230, 3: 61, 4: Trx. In (**B**), Seq1 indicates the starting position of NAIS230 and NAIS291, and Seq2 indicates the starting position of NAIS61.

In this study, bacterial Trx was used as a model antigen. Typically, Trx is known to be used to stabilize recombinant proteins expressed in neighboring genes because of its protective effect against oxidative stress and reactive oxygen. NAIS could be stably expressed in the Trx expression vector pBAD-DEST49, while it was also stably expressed in the pE-SUMO (Small Ubiquitin-like Modifier) pro Amp vector, which does not express Trx. The NAIS redesigned in this study is based on the protein reported by Sasaki et al. [11,13] and is derived from a bacterial outer membrane protein with the predicted regions of MHC (Major Histocompatibility Complex)-I and -II binding sites deleted (Figure 1). NAIS is a protein composed of 230 and 61 amino acids that share the first 53 amino acid sequences at the N-terminus. For comparative analysis, full-length NAIS291, NAIS230, and NAIS61 proteins fused with Trx and 6× His tag and Trx-fused 6× His tag were used as antigen variants for intranasal immunization (Figure 2). The cytotoxicity of NAIS toward to L929 cells determined by lactate dehydrogenase (LDH) assay was similar to that of BSA at 0.1–10 μM, indicating the low cytotoxicity of NAIS (Figure S1).

### 2.2. Antibody Titers Against Trx

**Figure 3:**
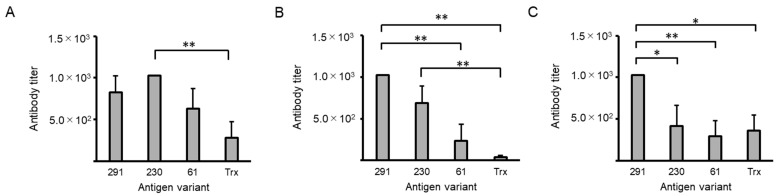
Changes in thioredoxin (Trx)-specific antibody titers in sera and bronchoalveolar lavage fluids (BALFs) determined by ELISA. (**A**) Serum IgG; (**B**) serum IgA; (**C**) BALF IgA. A statistical analysis was carried out using a generalized linear model. ** *p* < 0.01, * *p* < 0.05. Intranasal immunization with antigen variant was carried out at 14-day intervals three times. All sample sera or BALFs were confirmed to show higher antibody titers than untreated control sera or BALFs. The antibody titer on the vertical axis indicates the dilution factor of the sample.

A generalized linear model (GLM) analysis revealed significant differences in all antibody titers (*p* < 0.01, Figure 3), and serum IgG showed significantly higher anti-Trx antibody titers in the group immunized intranasally with NAIS230-fused Trx (230) than in the Trx group (*p* < 0.01, Figure 3A). In serum IgA, anti-Trx antibody titers obtained from mice immunized intranasally with NAIS291-fused Trx (291) were significantly higher than those of NAIS61-fused Trx (61) and Trx alone (*p* < 0.01, Figure 3B), and the antibody titer of 230 was also higher than that of Trx (*p* < 0.01). For bronchoalveolar lavage fluid (BALF) IgA, the anti-Trx antibody titers obtained after immunization with 291 were significantly higher than those obtained after immunization with all other antigens (230 and Trx: *p* < 0.05, 61: *p* < 0.01; Figure 3C). In a comparative analysis of the statistics, one-way analysis of variance (ANOVA) revealed equal variances in serum IgG (*p* < 0.05), serum IgA (*p* < 0.01), and BALF IgA (*p* < 0.01); the anti-Trx serum IgA antibody titers of 291 were significantly higher than those of Trx alone by post hoc Tukey’s test (*p* < 0.01, Figure S2).

### 2.3. Antibody Titers Against NAIS

**Figure 4:**
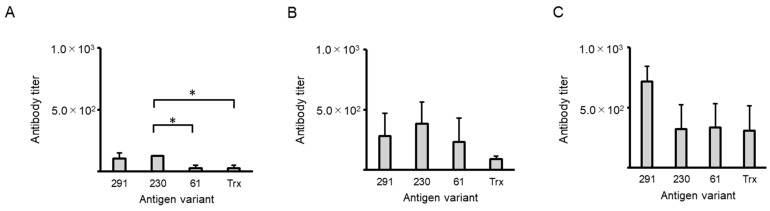
Changes in nasal immuno-inducible sequence (NAIS)-specific antibody titers in sera and bronchoalveolar lavage fluids (BALFs) determined by ELISA. (**A**) Serum IgG; (**B**) serum IgA; (**C**) BALF IgA. A statistical analysis was carried out using a generalized linear model. * *p* < 0.05. Intranasal immunization with antigen variant was carried out at 14-day intervals three times. All sample sera or BALFs had higher antibody titers than untreated control sera or BALFs. The antibody titer on the vertical axis indicates the dilution factor of the sample.

When immunized with NAIS fusion antigens, antigen recognition may be impaired if antibodies are produced against NAIS alone. Therefore, anti-NAIS antibody titers were explored (Figure 4), and the GLM test showed significant differences between peptides only for serum IgG (*p* < 0.05), while the post hoc test showed that serum IgG obtained from 230 immunized mice was significantly higher than that from 61- or Trx-immunized mice (*p* < 0.05, Figure 4A).

### 2.4. Comparison of Anti-Trx and Anti-NAIS Antibody Titers

**Figure 5:**
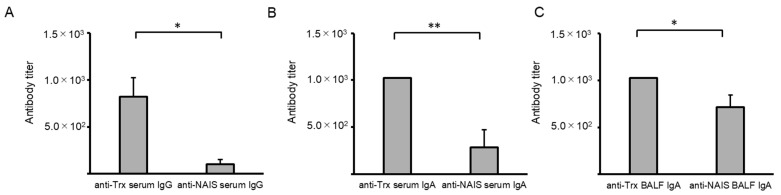
Comparison of thioredoxin (Trx)- and nasal immuno-inducible sequence (NAIS)-specific antibody titers in sera and bronchoalveolar lavage fluids (BALFs) obtained from NAIS291-fused Trx protein immunized mice. (**A**) Serum IgG; (**B**) serum IgA; (**C**) BALF IgA. A statistical analysis was carried out using a *t*-test. ** *p* < 0.01; * *p* < 0.05. Intranasal immunization with antigen variant was carried out at 14-day intervals three times. All sample sera or BALFs were confirmed to be shown higher antibody titers than untreated control sera or BALFs. The antibody titer on the vertical axis indicates the dilution factor of the sample.

To explore antibody production against NAIS and Trx, sera and BALFs from mice immunized with the full-length Trx fusion protein 291 were compared (Figure 5). Trx antibody titers were significantly higher than those of NAIS at for serum IgG and BALF (*p* < 0.05) as well as serum IgA (*p* < 0.01). These results indicate that immune cells target Trx rather than NAIS.

## 3. Discussion

With respect to the amino acid sequences used in the MP3 protein and NAIS, in vitro and in vivo studies have shown adhesion to the extracellular matrix [11,13], but no significant functional sequences have been found in the protein database (http://pfam.xfam.org/ accessed on 25 November 2024). Therefore, it was inferred that the sequences in NAIS have unknown functions.

In this study, Trx was used as a model antigen and fused with NAIS, which was developed by eliminating the predicted antigenicity site of the original bacterial protein. The Trx used in this study is derived from *E. coli* strain K12 and is responsible for promoting solubility by promoting the reduction of disulfide bonds formed by cysteine residues in recombinant protein. Since NAIS is stably expressed even with SUMO tags, Trx itself is not expected to have a significant effect on NAIS. In particular, Trx itself was used as a model antigen for protein expression in prokaryotes because it is potent and easily expressed. Although Trx is known to be present in all organisms, it is unlikely to affect antibody production in mice due to low amino acid homology between prokaryotic and eukaryotic cells. In this study, Trx from *E. coli* was placed at the N-terminus for efficient expression, but when fusing nonprokaryotic proteins with NAIS, prokaryotic codon usages at the N-terminus would be more efficient for expression in the *E. coli* system.

Multiple intranasal immunization with fusion protein of NAIS was observed to lead to elicit specific antibodies in mice. These results have unique implications for intranasal vaccination. In brief, intranasal vaccination with Trx fused with full-length NAIS elicited IgA production more effectively than Trx alone, and anti-NAIS antibodies were produced less effectively than the target antibody in mice. Furthermore, the humoral immunity elicited by NALT via the intranasal route induces the production of systemic secretory antibodies. Several unique bacterial proteins act as immunomodulators [14,15]. These results suggest that NAIS may be responsible for immunomodulation and assist in the immunostimulation of fused target antigens, such as bacterial proteins.

The activation of innate immunity, including antigen-presenting cells and Toll-like receptors (TLRs), is essential for vaccines to be effective, and adjuvants play a role in vaccine components [16]. Classical vaccines use aluminum hydroxide and aluminum phosphate, which induce IgE; thus, classical adjuvants should be improved to increase safety [17]. A recently developed vaccine design approach is a viral vector vaccine, in which a specific antigen is inserted into the genome of a modified virus that can infect host cells; the antigen, along with the viral particles, is recognized by the immune system as a pathogenic signal, inducing an adaptive immune response. However, this system has a low booster effect, that is, immune recognition of viral particles and transport carriers [17,18,19]. As the antibody titer of NAIS was lower than that of Trx at up to three doses, NAIS may also have a superior booster effect. Among the antibodies against NAIS, IgA from the BALF showed the highest antibody titer, indicating that NAIS-fused antigen inoculation promoted B cell differentiation into IgA plasma cells.

Recently, various substrates have been developed as adjuvants and antigen carriers for intranasal vaccines [1,2,6,8,20]. Among these, saponin-based adjuvants, including QS-21 and ISCOMATRIX (IMX), are functionally evident [8,21,22]. QS-21 binds to cell-surface lectins via its carbohydrate domain, leading to antigen uptake by APCs and stimulation of specific cytokines activating cellular and/or humoral responses [23]. In a recent report, QS-21 was added to a vaccine formulation under development against *Trichuris muris* infection and administered intranasally as a dual-adjuvanted vaccine to enhance both humoral and cellular immunity against *T*. *muris* infection in the mouse intestinal tract [24]. IMX is a 40 nm particle composed of saponins and other substances that can elicit influenza virus-specific sIgA in the intestinal tract, alveoli, and nasal cavity when administered intranasally as an adjuvant with an inactivated influenza vaccine [22]. It has a negatively charged surface, which may limit its binding to neutral or negatively charged hydrophilic antigens [23]. These saponin-based adjuvants have potent immuno-inducing effects and versatility for intranasal inoculation but require the step of conditioning them to be separate from the antigen.

Although bacterial toxins, including heat-labile *E. coli* and cholera toxins, have been developed experimentally as immunomodulator adjuvants, the early stage of adjuvants has not been used clinically because of their pathogenicity and safety as intranasal vaccine adjuvants in humans [25]. However, various modifications of bacterial enterotoxins have been made and clinical studies have been conducted, and although no serious adverse reactions have occurred, moderate symptoms such as nausea and diarrhea have been reported for oral vaccines and abnormal injection site symptoms for intramuscular injection [26]. Although there are no adverse reactions such as severe facial paralysis, nasal inoculation with bacterial toxins will have to be pursued for further safety [26]. The cytotoxicity assay of NAIS revealed a low equivalence to BSA, and NAIS was not notably pathogenic toward mammalian cells. Bacterial cell walls and LPS are not immunogenic and enhance immune responses but activate TLRs that mediate the host immune defense system [27]. When utilizing this method, it is necessary to develop a method to remove bacteria LPS while maintaining the protein level, since the effect of LPS on immune activity, which is one of the issues in this study, is unavoidable. Bacterial proteins with such functions may be detrimental to the host during natural bacterial infections. However, these proteins may have assistive functions in terms of immune stimulation. Bacterial polypeptides with unique functions have been used as immune stimulation components in therapies for diseases other than infectious diseases [28,29]. These results indicate that NAIS may be useful as not only a vaccine for infectious diseases, but also a treatment for cancer, allergies, and other conditions requiring immune stimulation. Although NAIS may act based on a mechanism similar to that of bacterial components, this mechanism needs to be elucidated. This study did not measure cellular immunity or the effect of infection prevention by experimental infection, which will be an issue for future studies.

## 4. Materials and Methods

### 4.1. Intranasal Immunization Materials

In previous study, we designed an intranasal vaccine that retained the antigenicity of MP3 properties of the original protein, PnxIIIA (GenBank accession no. BAJ09609.1) [13]. In this study, we re-designed a protein with low molecular weight and low antigenicity while retaining the function of the original MP3 protein (Figure 1). In brief, the protein structure was modified to remove a large number of predicted MHC-I and -II binding sites in the PnxIIIA and MP3 proteins in order to reduce the number of antigen epitope sites.

Figure 2 shows the primary structure of the intranasally immunized materials (A) and the amino acid sequence of the NAIS (B). NAIS is a 291 amino acid residue polypeptide comprising two repeating sequences, without being responsible for pathogenicity [11] and antigenicity removed by epitope prediction [30,31]. NAIS originates from an outer membrane protein identified in the genus *Rodentibacter* [12,13]. To explore its immunoinducibility, a fusion protein expressing Trx at the N-terminus and a 6× His tag at the C-terminus were produced using the pBAD-DEST49 and BL21-AI *E. coli* protein expression systems (Thermo Fisher Scientific, Waltham, MA, USA), respectively. In brief, *E. coli* BL21-AI carrying pBAD based plasmid were cultured overnight at 37 °C in LB (Luria–Bertani) broth containing 100 μg/mL ampicillin (final concentration) with shaking, then added to 30 mL fresh LB broth at 1% and further cultured for 2 h at 37 °C. After adding 0.002% L-arabinose (final concentration), the bacteria were cultured overnight at room temperature (25 °C). The cells were then centrifuged at 5000× *g* for 10 min, suspended in tris-buffered saline (TBS, pH7.4) containing 1 mM imidazole, and disrupted using a sonicator, model THU-80 (AS ONE, Osaka, Japan). The soluble proteins were then purified using the Dynabeads His-Tag Isolation and Pulldown kit (Veritas, Tokyo, Japan) according to the manufacturer’s instructions. To compare the indigenous NAIS repeat sequences (Figure 2B), the immunoinducibility of the Trx fusion proteins with NAIS230 and NAIS61 was examined (Figure 2A). Bradford reagents (Merck, Rahway, NJ, USA) were used to measure protein concentrations and to adjust protein concentrations for subsequent testing. To measure antibody titers against NAIS alone, recombinant NAIS protein was produced using the pE-SUMOpro Amp vector (Lifesensors, Malvern, PA, USA) and *E. coli* BL21 (DE3) (New England Biolabs, Ipswich, MA, USA). In brief, the full-length NAIS was inserted into the *Bsa*I and *Sal*I sites, expressed as described above using isopropyl β-D-thiogalactopyranoside, then treated with SUMO protease 1 (Lifesensors) to remove the SUMO tag according to manufacturer’s instructions. Finally, NAIS treated with Dynabeads (Veritas) was used for an enzyme-linked immunosorbent assay (ELISA).

### 4.2. Cytotoxicity Assay of NAIS Toward L929 Cells

To determine the cytotoxicity of NAIS, an LDH assay was performed using the mouse fibroblast-like cell line L929 (RCB2619). In brief, L929 cells were adjusted to 3000 cells/well (100 μL 3.0 × 10^4^/mL) and diluted with RPMI1640 (Fujifilm wako pure chemical, Osaka, Japan) containing 10% FBS medium (Biowest, Nuaillé, France) to final concentrations of 10 μM, 1 μM, and 0.1 μM BSA and NAIS. After 24 h of incubation, the cytotoxicity was calculated using an LDH cytotoxicity assay kit (Nacalai Tesque, Kyoto, Japan). For the LDH assay, the values are shown as the average of four replicates.

### 4.3. Intranasal Immunization of NAIS Fused Protein in Mice

**Figure 6:**
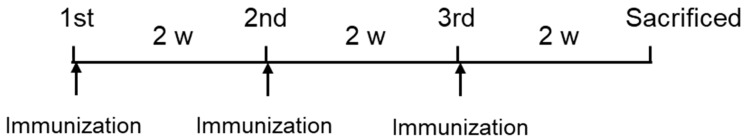
Experimental schedule for intranasal immunization of nasal immuno-inducible sequence (NAIS)-fused protein in BALB/c mice. There were five test groups: Groups 1–3 were intranasally immunized with three times for every two weeks 20 μL 3 μM NAIS291-, NAIS230-, and NAIS61-fused thioredoxin (Trx) and 6× His tag, respectively; Group 4 was immunized intranasally with 20 μL 3 μM Trx-fused 6× His tag alone; and a control group was untreated. Five mice were used per group. The animals were euthanized by isoflurane inhalation two weeks after the third immunization. Serum and bronchoalveolar lavage fluid were collected and analyzed. Groups 1 and 2 used 7-week-old wild-type BALB/c female mice (Charles River Japan, Yokohama, Kanagawa, Japan), while Groups 3–4 and the control used 8-week-old mice of same strain. All mice were maintained under specific pathogen-free (SPF) conditions and used in accordance with the experimental animal guidelines of Institutional Animal Care and Use Committee (IACUC) of Juntendo University under accession no. 2023009 and 2023010.

The experimental schedule for intranasal immunization of BALB/c mice with the NAIS-fused protein is shown in Figure 6. There were five test groups with five mice per group: Groups 1–3 were immunized intranasally with 20 μL 3 μM NAIS291-, NAIS230-, and NAIS61-fused Trx and 6× His tag, respectively; Group 4 was immunized intranasally with 20 μL 3 μM Trx-fused 6× His tag alone; and a control group was untreated. Intranasal immunization was performed three times every two weeks, and euthanasia was performed two weeks after the third immunization by inhalation of isoflurane. Serum and BALF were collected as described in a previous report [32] and analyzed. Groups 1 and 2 used 7-week-old wild-type BALB/c female mice (Charles River Japan, Yokohama, Kanagawa, Japan), while Groups 3–4 and the control used 8-week-old mice of the same strain. All mice were maintained under specific pathogen-free (SPF) conditions and used in accordance with the experimental animal guidelines of the IACUC of Juntendo University under accession numbers 2023009 and 2023010.

### 4.4. ELISA

ELISA was used to determine collagen-binding ability and antibody titers. To determine collagen-binding ability, collagen type IV coating 96-well plate (Coning, Bedford, MA, USA) was used. The method is the same as in the previous report [11], and HRP-conjugated HisProbe (Thermo Fisher Scientific, Waltham, MA, USA) was used for quantifying plate-binding MP3 and NAIS protein. The measurements were performed in triplicate, and the average absorbance values (A600) are shown in results.

For serum and BALF samples, anti-Trx or anti-NAIS antibody titers were determined by ELISA using the following methods: 96-well microtiter plates were coated with 0.5 μg/mL Trx-fused 6× His tag or NAIS and incubated overnight at 4 °C. Subsequently, the plates were blocked with protein-free blocking buffer (Thermo Fisher Scientific) for 2 h, and serially diluted serum or BALF samples were incubated in each well for 1 h. The plates were washed three times with phosphate-buffered saline with Tween 20 (PBS-T), and 1:3000 horseradish peroxidase (HRP)-conjugated Affinipure goat anti-mouse IgG (Proteintech, Rosemont, IL, USA) or IgA (Proteintech) diluted in PBS-T was then added to each well, followed by incubation for 1 h. Subsequently, the plates were washed three times with PBS-T and developed using 3,3′,5,5′-tetramethylbenzidine (TMB) solution (Atto, Tokyo, Japan). The IgG and IgA titers were determined by comparing the absorbance measured with a model Spectra Max 190 (Molecular Devices, San Jose, CA, USA) to that of untreated control serum or BALF. In this procedure, the titer was set to 1024 when the dilution was greater than 1024-fold. All sample sera or BALF samples had higher antibody titers than the untreated control sera or BALFs. The antibody titer was measured once for each animal, and when the absorbance was less than the mean of control, dilution factor was used as antibody titer. Also, if the absorbance was less than the average of control in two consecutive steps, the smaller dilution factor was used as results. All values are presented as the results section as mean ± standard error of the mean (SEM).

### 4.5. Statistical Analysis

For statistical analysis of the results obtained from the antibody titers, a GLM was employed. For the analysis of Trx-specific antibody titers, one-way ANOVA was performed to compare statistical analyses and the results are shown in the Supplemental Material. For comparison of Trx- and NAIS-specific antibody titers in the sera and BALF obtained from NAIS291-fused Trx-vaccinated animals, a *t*-test was employed. Differences were considered significant for *p* < 0.05 in the antibody titer assay.

## 5. Conclusions

In BALB/c mice, intranasal immunization with 291 amino acids of NAIS-fused Trx induced higher IgA antibody production than with Trx alone. These results suggest that NAIS has an immunostimulatory effect against the antigen and less antigenicity of NAIS itself compared with fused antigen. The results also indicate that NAIS may provide a new approach to mucosal immunity and facilitate the development of safe and effective intranasal vaccine systems.

## Data Availability

The original contributions presented in the study are included in the article/Supplementary Material; further inquiries can be directed to the corresponding author.

## Supplementary Materials

The following supporting information can be downloaded at: https://www.mdpi.com/article/10.3390/ijms252312828/s1.

## References

[1] Nakahashi-Ouchida R., Fujihashi K., Kurashima Y., Yuki Y., Kiyono H. (2023). Nasal vaccines: Solutions for respiratory infectious diseases. Trends Mol. Med..

[2] Kehagia E., Papakyriakopoulou P., Valsami G. (2023). Advances in intranasal vaccine delivery: A promising non-invasive route of immunization. Vaccine.

[3] Carter N.J., Curran M.P. (2011). Live attenuated influenza vaccine (FluMist^®^; Fluenz™): A review of its use in the prevention of seasonal influenza in children and adults. Drugs.

[4] Sun K., Ye J., Perez D.R., Metzger D.W. (2011). Seasonal FluMist Vaccin Induces Cross-React T Cell Immun H1N1 2009 influenza and secondary bacterial infections. J. Immunol..

[5] Sano K., Ainai A., Suzuki T., Hasegawa H. (2018). Intranasal inactivated influenza vaccines for the prevention of seasonal influenza epidemics. Expert. Rev. Vaccines.

[6] Thompson A.L., Staats H.F. (2011). Cytokines: The future of intranasal vaccine adjuvants. Clin. Dev. Immunol..

[7] Odelram H., Granström M., Hedenskog S., Duchén K., Björkstén B. (1994). Immunoglobulin E and G responses to pertussis toxin after booster immunization in relation to atopy, local reactions and aluminium content of the vaccines. Pediatr. Allergy Immunol..

[8] Fernández-Tejada A., Chea E.K., George C., Pillarsetty N., Gardner J.R., Livingston P.O., Ragupathi G., Lewis J.S., Tan D.S., Gin D.Y. (2014). Development of a minimal saponin vaccine adjuvant based on QS-21. Nat. Chem..

[9] Coughlan L., Kremer E.J., Shayakhmetov D.M. (2022). Adenovirus-based vaccines-a platform for pandemic preparedness against emerging viral pathogens. Mol. Ther..

[10] Filipić B., Pantelić I., Nikolić I., Majhen D., Stojić-Vukanić Z., Savić S., Krajišnik D. (2023). Nanoparticle-Based Adjuvants and Delivery Systems for Modern Vaccines. Vaccines.

[11] Sasaki H., Ishikawa H., Kojima K., Itoh M., Matsumoto T., Itoh T., Hosomi O., Kawamoto E. (2013). Intranasal immunization with a non-adjuvanted adhesive protein descended from *Pasteurella pneumotropica* and its preventive efficacy against opportunistic infection in mice. Vaccine.

[12] Sasaki H., Kawamoto E., Tanaka Y., Sawada T., Kunita S., Yagami K. (2009). Identification and characterization of hemolysin-like proteins similar to RTX toxin in *Pasteurella pneumotropica*. J. Bacteriol..

[13] Sasaki H., Ishikawa H., Sato T., Sekiguchi S., Amao H., Kawamoto E., Matsumoto T., Shirama K. (2011). Molecular and virulence characteristics of an outer membrane-associated RTX exoprotein in *Pasteurella pneumotropica*. BMC Microbiol..

[14] Johnson A.J., Kennedy S.C., Lindestam Arlehamn C.S., Goldberg M.F., Saini N.K., Xu J., Paul S., Hegde S.S., Blanchard J.S., Chan J. (2017). Identification of Mycobacterial RplJ/L10 and RpsA/S1 Proteins as Novel Targets for CD4+ T Cells. Infect. Immun..

[15] McSorley S.J., Ehst B.D., Yu Y., Gewirtz A.T. Bacterial flagellin is an effective adjuvant for CD4+ T cells in vivo. J. Immunol..

[16] Ishii K.J., Uematsu S., Akira S. (2006). ‘Toll’ gates for future immunotherapy. Curr. Pharm. Des..

[17] Aguilar J.C., Rodríguez E.G. (2007). Vaccine adjuvants revisited. Vaccine.

[18] Pasquale A.D., Preiss S., Silva F.T.D., Garçon N. (2015). Vaccine Adjuvants: From 1920 to 2015 and Beyond. Vaccines.

[19] Gregory A.E., Titball R., Williamson D. (2013). Vaccine delivery using nanoparticles. Front. Cell Infect. Microbiol..

[20] Bernal A.M., Sosa F.N., Todero M.F., Montagna D.R., Vermeulen M.E., Fernández-Brando R.J., Ramos M.V., Errea A.J., Rumbo M., Palermo M.S. (2023). Nasal immunization with H7 flagellin protects mice against hemolytic uremic syndrome secondary to *Escherichia coli* O157:H7 gastrointestinal infection. Front. Cell Infect. Microbiol..

[21] Detienne S., Welsby I., Collignon C., Wouters S., Coccia M., Delhaye S., Van Maele L., Thomas S., Swertvaegher M., Detavernier A. (2016). Central role of CD169+ lymph node resident macrophages in the adjuvanticity of the QS-21 component of AS01. Sci. Rep..

[22] Coulter A., Harris R., Davis R., Drane D., Cox J., Ryan D., Sutton P., Rockman S., Pearse M. (2003). Intranasal vaccination with iscomatrix adjuvanted influenza vaccine. Vaccine.

[23] Pearse M.J., Drane D. (2005). ISCOMATRIX adjuvant for antigen delivery. Adv. Drug Deliv. Rev..

[24] Wei J., Hegde V.L., Yanamandra A.V., O’Hara M.P., Keegan B., Jones K.M., Strych U., Bottazzi M.E., Zhan B., Sastry K.J. (2022). Mucosal vaccination with recombinant *Tm*-WAP49 protein induces protective humoral and cellular immunity against experimental trichuriasis in AKR mice. Front. Immunol..

[25] Lycke N., Lebrero-Fernández C. (2018). ADP-ribosylating enterotoxins as vaccine adjuvants. Curr. Opin. Pharmacol..

[26] Crothers J.W., Norton E.B. (2023). Recent advances in enterotoxin vaccine adjuvants. Curr. Opin. Immunol..

[27] Patel R.B., Ye M., Carlson P.M., Jaquish A., Zangl L., Ma B., Wang Y., Arthur I., Xie R., Brown R.J. (2019). Development of an in situ cancer vaccine via combinational radiation and bacterial-membrane-coated nanoparticles. Adv. Mater..

[28] Zhao X., Zhao R., Nie G. (2022). Nanocarriers based on bacterial membrane materials for cancer vaccine delivery. Nat. Protoc..

[29] Gao X., Feng Q., Wang J., Zhao X. (2022). Bacterial outer membrane vesicle-based cancer nanovaccines. Cancer Biol. Med..

[30] Peters B., Sette A. (2005). Generating quantitative models describing the sequence specificity of biological processes with the stabilized matrix method. BMC Bioinform..

[31] Nielsen M., Lundegaard C., Lund O. (2007). Prediction of MHC class II binding affinity using SMM-align, a novel stabilization matrix alignment method. BMC Bioinform..

[32] Kunimine S., Takai T., Kamijo S., Maruyama N., Kimitsu T., Masutani Y., Yoshimura T., Suchiva P., Shimizu S., Ogawa H. (2021). Epicutaneous vaccination with protease inhibitor-treated papain prevents papain-induced Th2-mediated airway inflammation without inducing Th17 in mice. Biochem. Biophys. Res. Commun..

