# Supplementary material for: Intranasal Immunization with Nasal Immuno-Inducible Sequence-Fused Antigens Elicits Antigen-Specific Antibody Production
Source: Int J Mol Sci. 2024 Nov 28;25(23):12828. doi: 10.3390/ijms252312828 (PMC11641730; doi:10.3390/ijms252312828)
Supplement: Supplementary file 1 [file ijms-25-12828-s001.zip › ijms-3317466-supplementary.pdf]

Supplementary figures

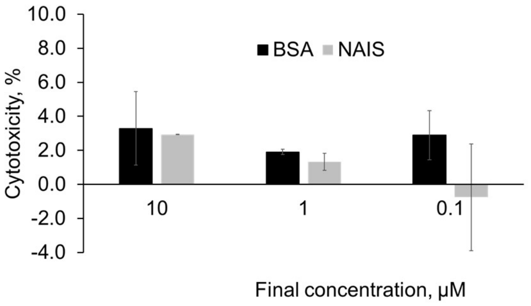

Figure S1. Cytotoxicity assay of NAIS in L929 cells as determined by lactate dehydrogenase (LDH) assay. Mouse fibroblast-like cell line L929 cells were adjusted to 3,000 cells/well (100 μL 3.0×10^4/mL) and diluted with DMEM to final concentrations of 10 μM, 1 μM, and 0.1 μM BSA and NAIS. After 24 hours of incubation, the cytotoxicity % was calculated using the LDH cytotoxicity assay kit (Nacalai tesque, Kyoto, Japan).

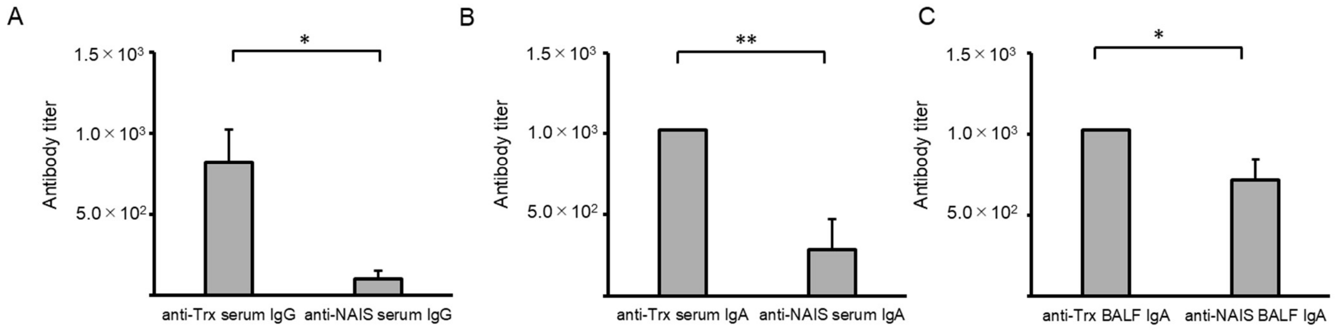

Figure S2. Changes in Trx-specific antibody titers in sera and BALFs determined by ELISA. (A) Serum IgG; (B) Serum IgA; (C) BALF IgA verified with a one-way ANOVA post hoc Turkey's test. A one-way ANOVA revealed equal variances in serum IgG (P<0.05), serum IgA (P<0.01), and BALF IgA (P<0.01). Post hoc test showed asterisks if there were significant differences between the antigen variants. ** P<0.01. Intranasal immunization with antigen variant was carried out at 14-day intervals for three times. All sample sera or BALFs had higher antibody titers than the untreated control sera or BALFs. The antibody titer on the vertical axis indicates the dilution factor of the sample.
